# Short- and long-term changes in neurological, behavioural, and blood biomarkers following repeated mild traumatic brain injury in rats—potential biological sex-dependent effects

**DOI:** 10.3389/fnmol.2025.1488261

**Published:** 2025-01-29

**Authors:** Rodrigo Moraga-Amaro, Oscar Moreno, Jordi Llop, Marion Bankstahl, Jens P. Bankstahl

**Affiliations:** ^1^Department of Nuclear Medicine, Hannover Medical School, Hanover, Germany; ^2^CIC biomaGUNE, Basque Research and Technology Alliance (BRTA), San Sebastián, Spain; ^3^Institute for Laboratory Animal Science, Hannover Medical School, Hanover, Germany; ^4^Department of Biological Sciences and Pathobiology, Institute of Pharmacology, University of Veterinary Medicine Vienna, Vienna, Austria

**Keywords:** rmTBI, CTE, concussion, sex differences, tau, NSE

## Abstract

**Introduction:**

Chronic traumatic encephalopathy (CTE) is a progressive neurodegenerative disease resulting from repeated mild traumatic brain injuries (rmTBI). The necessity for diagnosis of CTE, which can so far only be confirmed after post-mortem, is a pressing need. New approaches to early diagnose this disease are crucial to facilitate the translation of novel treatment strategies to the clinic. Several studies have found suitable candidate biomarkers, but the results are not straightforward. As biological sex is suggested to be a major confounding factor, we explored how sex influences behavioural and candidate blood biomarkers during CTE-like progression following experimental rmTBI.

**Methods:**

To induce CTE-like development, we subjected male and female rats to three mTBIs at a 5-day interval. We then monitored and analysed differences in neurological, behavioural, and physiological parameters up to 12 weeks after the injuries—both by sex and grouped—and underwent further analysis using generalised estimated equation (GEE). To determine long-term changes in tau aggregation as a hallmark of CTE, we used [^18^F]-florzolotau (florzolotau) autoradiography in brain slices.

**Results:**

Both short-term weight gain and time-to-right after rmTBI were increased in grouped animals, with male rats showing more prominent changes. The neurological state was impaired after each mTBI and still 12 weeks later, independent of the sex. A protracted anhedonic-like behaviour due to rmTBI was found at the group level only at week 2 but remained continuously present in male rats. While spatial memory was not impaired, male rats showed increased anxiety-like behaviour. Moreover, neuron-specific enolase (NSE) was elevated in the blood 1 day after rmTBI, but only in females. On the contrary, blood p-tau was increased 3 days after rmTBI only in males. In addition, male rats showed significantly increased florzolotau binding in the brain after 12 weeks, suggesting brain contusion causes increased tau aggregation. Interestingly, brain neurofibrillary tangles (NFTs) at 12 weeks after rmTBI showed a strong correlation with the neurological state at 1 day after rmTBI.

**Discussion:**

Taken together, our findings suggest that male rats may be more susceptible to short-and long-term consequences of rmTBI in the applied model. These sex differences should be considered when translating preclinical biomarker candidates to the clinic. Understanding these differences could guide the diagnosis and treatment of CTE in a personalized manner, offering hope for more effective treatments in the future.

## 1 Introduction

In recent years, clinical observations have raised awareness of the effects of repeated mild traumatic brain injuries (rmTBI), also referred to as concussions, since they can lead to early-age dementia and chronic neurodegeneration (Graham and Sharp, 2019). This pathology, known as chronic traumatic encephalopathy (CTE), is strongly associated with, but not restricted to, (contact) sports participation and exposure to explosive blast (McKee et al., 2013). A study performed in 2017 showed a high incidence of CTE, with approximately 88% of former US football players diagnosed with CTE (Mez et al., 2017). In general, concussions cause neurological impairments due to microscopically minor injuries, including the neurovascular unit, resulting in further neurodegeneration (Pearn et al., 2017). CTE’s neuropathological hallmark is a widespread and increasing deposition of neurofibrillary tangles (NFT) in the brain, mostly composed of phosphorylated tau (p-tau) (Stern et al., 2011; Saing et al., 2012). For this reason, CTE is classified as a tauopathy (Juan et al., 2022).

A single mTBI can cause short-term (acute) symptoms that may resolve within days, weeks, or months after the concussion, a condition referred to as post-concussive syndrome (PCS) (Minen et al., 2016). However, single mTBI effects often remain unnoticed since, in some cases, there is a lack of immediate symptoms. In this regard, persistent underlying physiological damage due to injury, such as axonal damage, is still present (Hofman et al., 2001). With subsequent rmTBI events, this damage increases over time but remains silent, thus making CTE very difficult to diagnose in its early stages (Iverson et al., 2019). While studies already investigated possible biomarkers related to neurodegeneration, there is a high variability in the results (Agoston et al., 2022; Mavroudis et al., 2022; Halicki et al., 2023). In this context, the complexity of finding early biomarkers for CTE might be complicated by the biological sex factor. Biological sex is based on the presence of X and Y chromosomes, being the most common sexes, female (XX) and male (XY) (Bale, 2019). This genetic difference has been shown to have an influence on different disease incidences (Pinares-Garcia et al., 2018), onset, pathology, and treatment response, with sex hormones being mainly responsible for these observations (Morris et al., 2004). In fact, some research has already proven the importance of sex differences in several brain diseases (DuMont et al., 2023), although its molecular basis is not yet completely understood. A recent scoping review has shown that the incidence of mTBI in contact sports is higher in female athletes, accompanied by worse outcomes and recovery when compared to men (Musko and Demetriades, 2023). Sex differences have also been found in biomarkers of patients who suffered mild or severe TBI. Studies of large cohort of trauma patients observed that the amount of the plasma biomarkers Glial Fibrillary Acidic Protein (GFAP) and Ubiquitin Carboxy-terminal Hydrolase-L1 (UCH-L1) as surrogate markers of brain injury was higher in males than in female individuals (Papa et al., 2023), whereas tau protein amounts were higher in the plasma of females (Clarke et al., 2021). In the same line, an increased cerebral blood flow was found in male patients following mTBI, correlating with a worse cognitive performance, which was not observed in females (Bai et al., 2019). A review of the effects of sex on mTBI biomarkers was done by Strathmann et al. (2014). Their observations support the notion that brain pathophysiology should be studied by taking factors, such as biological sex, into account. While contact sports used to be predominantly practiced by men, the increasing participation of women in this field also argues for the relevance of investigating sex differences in concussion-related pathology. While CTE data in women is very scarce, the first case of CTE in a former professional female footballer was recently reported (Suter et al., 2023).

In this study, a previously validated rat model of CTE-like pathology was used, consisting of three consecutive mTBI with a 5-day interval (McAteer et al., 2016). Using a combination of neurological tests, behavioural investigations, and targeted blood and brain analyses, we aimed to evaluate the pathophysiology of CTE-like pathology development in a longitudinal manner, focusing on sex differences in the biomarker changes.

## 2 Methods

### 2.1 Animals and study design

Animals and study design are reported according to the Animal Research: Reporting *In Vivo* Experiments (ARRIVE 2.0) Essential 10 guidelines. All experiments were conducted by the European Communities Council Directive 2010/63/EU, and approved by the local authorities (LAVES) under the protocol ID 20-3364. A total number of 16 Sprague–Dawley rats (RjHan:SD, Janvier, 13-weeks old, *n* = 8 males; *n* = 8 females) was divided into control animals (Sham; *n* = 4 males and *n* = 4 females) and experimental animals (rmTBI; *n* = 4 males and *n* = 4 females). When surgeries were performed, the age of the animals was 14 weeks. Animals arrived in cohorts of 4 (2 male and 2 female) and were randomly picked one by one and alternatively assigned to the sham or rmTBI group. Males and females were housed separately, but sham and rmTBI animals were housed together. Due to the study’s exploratory nature, no prior sample size calculation was performed. All animals underwent the same experimental procedures, except for the sham animals, which were exposed to the surgery but not to mTBI procedures. Habituation of the animals to the animal room and the experimenter was started at least 1 week before beginning the experimental procedures. During this habituation period, all animals were handled at least 3 times. All animals were pair-housed (1 sham and 1 mTBI rat per cage) in enriched (nesting material and wooden balls) individually ventilated biocontainment units (Scantainer Allentown, Scanbur, Denmark) under a 14/10-h light/dark cycle with *ad libitum* access to standard laboratory food pellets (Altromin 1324 TPF, Altromin, Germany), autoclaved tap water and standard laboratory conditions (temperature 21.5–23.0°C; humidity 45–55%). *A priori* inclusion criteria were not established. *A priori* exclusion criteria were specified for each outcome measurement and are stated in the respective sections. However, no animals or data points were excluded from this study.

**Figure 1:**
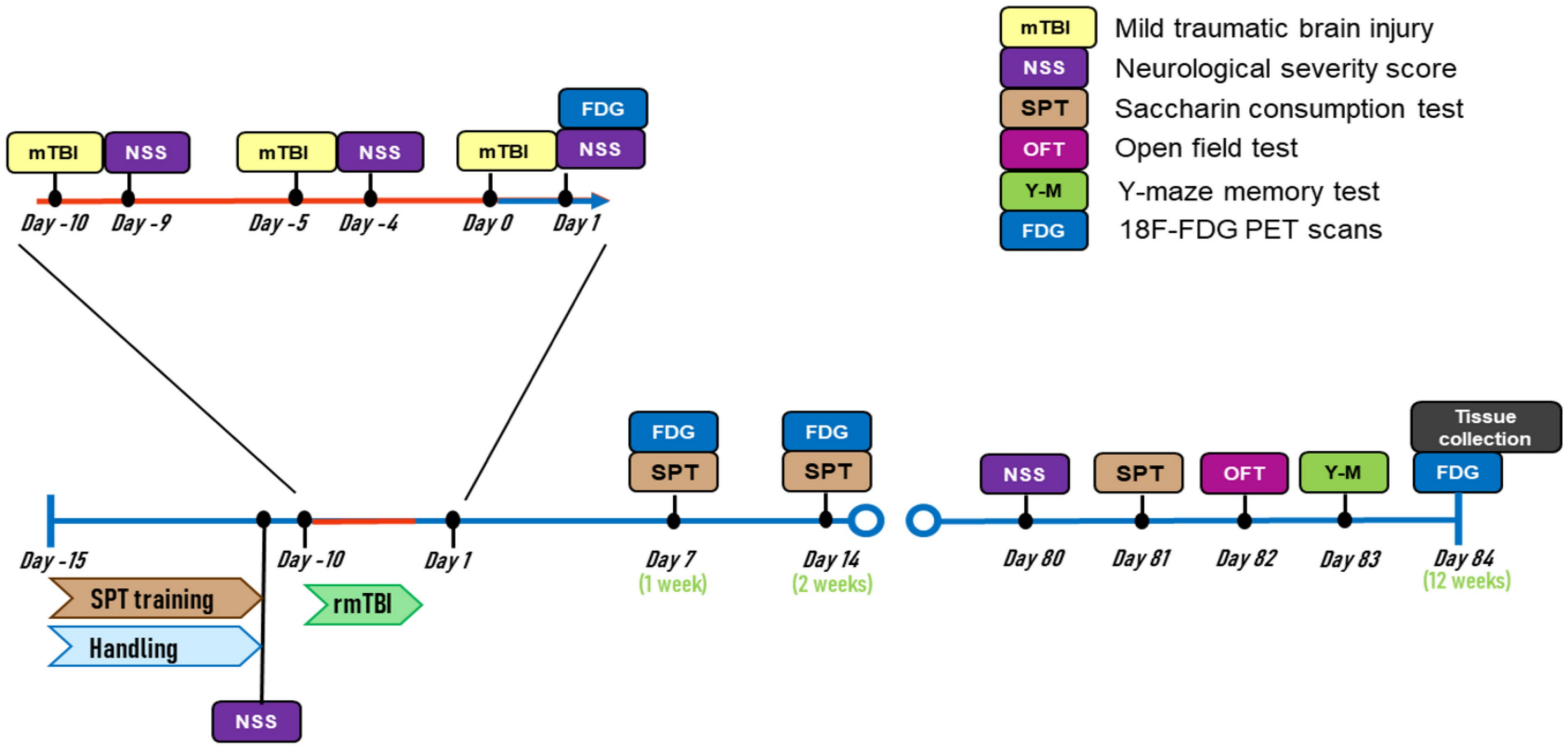
Experimental timeline.

A timeline of all experimental procedures is depicted in Figure 1. All animals were weighed on the morning before and after each sham/mTBI induction, and every 24 h for 2 consecutive days after surgery. Three sham/mTBI inductions were performed with a 5-day interval in between. A neurological severity score (NSS) was performed before the first mTBI (baseline), the day after each of the sham/mTBI surgeries, and 12 weeks after the last mTBI. A saccharin preference test (SPT) was used to measure anhedonic-like behaviour. SPT training was conducted before the sham/mTBI surgeries and subsequently performed 1, 2, and 12 weeks after the last sham/mTBI surgery. Plasma samples for blood biomarker quantification were obtained on days 1, 3, 7, 14, and 12 weeks after the end of surgeries. At the end of the experiments, an open field test (OFT) and a Y-maze test were used to measure anxiety-like behaviour and spatial memory. After behavioural experiments in week 12, animals were sacrificed, and brains were taken out. Brains were sliced for p-tau *in vitro* autoradiography using the [^18^F]-florzolotau (florzolotau) radiotracer. In this regard, acute effects of rmTBI were measured during the first 2 weeks after the last injury, while chronic, CTE-like effects of rmTBI were measured at 12 weeks post-injury.

In this study, “sex” and “injury” were analyzed as variables influencing the different outcomes. While “sex” refers to males and females, “injury” refers to sham-or rmTBI-subjected animals. Analysis of the effect of “injury” independent of sex (Level 1; sham vs. rmTBI), was performed with male and female animals merged (stated as “grouped”). The analysis of a combined effect of “injury x sex” (level 2; males: sham vs. rmTBI, females: sham vs. rmTBI) was performed by comparing rmTBI against sham in males and females independently. Since the majority of the outcome parameters were analysed at different time points, the analyses were performed comparing the interaction between ‘sex’, ‘injury’, and ‘time point’, except for parameters measured in the OFT, Y-maze, and autoradiography. This multifactorial analysis was applied as described in a previous study (Eyolfson et al., 2020).

### 2.2 Induction of rmTBI

RmTBI was induced using an impact acceleration model of diffuse traumatic injury as described before (Marmarou et al., 1994; McAteer et al., 2016), producing a 110-g average lineal acceleration force. Three mTBIs were performed, with a 5-day interval in between. For the surgery, anaesthesia was induced by 5% isoflurane in oxygen for up to 3 min (induction box), then maintained with an isoflurane concentration between 2 and 3% (nose mask). Heating pads were applied to maintain physiological body temperature and eye ointment was applied to prevent corneal dehydration. The fur on the rats’ heads was shaved, and local anaesthesia was applied cutaneous (2% tetracaine). After 5 min, an incision to the skin above the bregma and lambda area on the skull was made, and 0.25% bupivacaine solution was applied; after 3 min, a stainless-steel disc (10-mm diameter, 3-mm height) was adhered centrally between bregma and lambda on the interstitial tissue, using standard polyacrylamide adhesive (Pattex, Henkel, Germany). For mTBI induction, animals were placed in a standardized position on a foam bed (type-E bed foam) with a horizontal head position and the stainless-steel disc aligned with a plastic tube containing a brass weight (450 g). The brass weight was released through the tube from a height of 1.00 m, hitting directly on the steel disc. This procedure required an interruption of isoflurane supply of 10–15 s. After the hit, the animals’ noses were placed back into the anaesthesia mask, the metal disc was removed, and the incision was sutured after additional local application of 0.25% bupivacaine solution. Sham-operated animals received the identical treatment, except for the brass weight hit. After suturing, the isoflurane supply was stopped, animals were placed supine in a clean cage, and the time-to-right, starting with the first sign of regain of consciousness (hind-or forepaw movement), was recorded.

### 2.3 NSS

To assess changes in the neurological state of the animals across time, we used the revised NSS for rats described by Yarnell et al. (2016). This test evaluates 10 different responses with scores from 0 to 2, zero being a normal response, 1 being a partial response, and 2 being a complete impaired response. The evaluations were (1) general balance on a beam walk (1-m long, 2-cm wide, positioned at a height of 29 cm; measuring if animals could cross the beam, maintain balance or fall in 15 s), (2) landing test (measuring if rats land evenly on four paws or flat), (3) tail raise test (measuring paw extension and body twisting), (4) drag test (checking if rats are grasping trying to slow down the dragging), (5) righting reflex (measuring flipping reaction when rats are positioned on their back), (6) ear reflex (checking for flattening when touching with a cotton swap), (7) eye reflex (checking for eye blink when approaching with a cotton swap), (8) sound reflex (checking for a startling response to a hand clap), (9) tail reflex (presence of squeak when pressing the tail), and (10) paw flection reflex (checking of the limb withdrawal reflex after pressing the hindlimb paws). The experimenter was not blinded to the experimental groups.

### 2.4 SPT

The SPT measured longitudinal changes in anhedonic-like behaviour, as previously described (Moraga-Amaro et al., 2022), but using saccharin instead of glucose. Training was performed to adapt animals to a saccharin 0.1% solution consumption for four consecutive days. During both training and test sessions, animals were single-housed. The training consisted of a three-consecutive-day exposure to a single bottle with saccharin solution for 3 h during the light phase, followed by an overnight exposure to a choice between two bottles, one filled with normal drinking water and the other with the saccharin solution. SPTs were performed at 1, 2, and 12 weeks after rmTBI. SPTs consisted of an overnight exposure to a choice between a bottle filled with normal drinking water, and another containing saccharin solution. The amount of fluid consumption was measured by weighing the bottles, assuming that 1 g = 1 mL, and the percentage of saccharin solution over the total intake was calculated and used as an outcome parameter. The experimenter was not blinded to the experimental groups. Exclusion criteria included bottles being emptied by malfunctioning, but no data points were excluded.

### 2.5 OFT

The OFT was used to measure locomotion and anxiety-related behaviour, as described before (Moraga-Amaro et al., 2022). In brief, rats were placed inside a circular arena (90 cm diameter) for 5 min. OFT was performed in a separate quiet room, and light intensity was adjusted to 5 lux to enhance exploration of the arena. During OFT trials, the experimenter was outside the room, to avoid distractions of the animals. Parameters assessed to evaluate anxiety-like behaviour were the time animals spent in the centre of the arena (20 cm from the border was set as the diameter) and the number of times they crossed from the periphery to the centre. Locomotion was measured as the distance (in meters) travelled in the arena. Sessions were recorded with a digital camera (Redflexx, RC-300, Red Eagle IT, Germany) for further offline analyses. All analyses were performed using the ANY-maze v8.3 software (Ugo Basile srl. IT, Stoelting Europe, Ireland). The experimenter was not blinded to the experimental groups; however, data analysis was automatized. The exclusion criterion for the OFT was animals escaping from the setup, although no data sets were excluded.

### 2.6 Y-maze test

The Y-maze was used to measure long-term spatial memory changes (Jung et al., 2008). The Y-maze apparatus consisted of three arms (50 cm × 20 cm × 30 cm each) joined together with a 120° separation angle. Training and tests in the Y-maze apparatus were performed in the same room as the OFT, under the same light intensity, and with the experimenter outside the room. The training session consisted of placing the animals in the Y-maze, with one of the arms randomly closed. Animals were left to explore the two remaining open arms for 15 min. Visual clues were placed in the room to allow animals to use them as spatial references. The test session was performed on the same day, 6 h after the training. In the test session, animals were allowed to explore all three arms for 5 min. Time spent in each arm and the distance travelled were measured using the ANY-maze v8.3 software (Ugo Basile srl. IT). The percentage of time spent in the previously closed arm, compared to the total time (time spent in the novel arm (%)), and number of times the animal entered the novel arm were calculated and used as outcome parameters. The experimenter was not blinded to the experimental groups, but data analysis was automatized. The same exclusion criterion for the OFT was used for the Y-maze, but no animal dataset qualified for this criterion.

### 2.7 Plasma biomarker quantification

Plasma samples were obtained at days 1 and 3 1, 2, and 12 weeks after rmTBI, to measure possible changes in blood biomarkers. Approximately 300 μL of blood were collected from a lateral tail vein of isoflurane-anaesthetized rats in EDTA tubes (Microvette^®^ CB 300 K2E, Sarstedt, Germany) and immediately centrifuged at 5,000 *g* for 10 min at 4°C. Plasma was then collected and stored at −80°C. A commercially available enzyme-linked immunosorbent assay (ELISA) kit was used for the quantification of total free plasma p-tau and/or phosphorylated microtubule-associated phospho-tau (pMAPT; catalog n° E-EL-R1090NSE, Elabscience, Texas, USA) and neuron-specific enolase (NSE; catalog n° MBS262217, MyBioSource, California, USA) as an established marker for traumatic brain injury-related diseases (Stępniewska et al., 2024). We will refer to plasma “Pmapt and p-tau” as “p-tau” alone for practical purposes. Samples were diluted 1:5 using the “dilution buffers” from the kits, and quantification was performed according to the specifications given by the manufacturers. The ELISA plates were read at 450 nm using a plate reader (BGM, FLUOstar Optima, BGM Labtech, Germany), and plasma p-tau and NSE were calculated using the calibration curves.

### 2.8 Florzolotau synthesis

Florzolotau was synthetised using a TRACERlab FX_FN_ synthesis module (GE Healthcare) following a previously described method with minor modifications (Kawamura et al., 2021). The synthesis was performed without light to avoid the photoisomerization of both the precursor and radiotracer, except for specific steps, where monitoring was needed (e.g., HPLC injection). In brief, [^18^F]F^−^ was generated in a Cyclone 18/9 cyclotron (IBA RadioPharma Solution, Belgium) by proton irradiation of ^18^O-enriched water *via* the ^18^O(p,n)^18^F nuclear reaction, and trapped on a preconditioned Sep-Pak® Accell Plus QMA Light cartridge (Waters, Milford, MA, USA). [^18^F]F-was eluted from the cartridge with a mixture of aqueous potassium carbonate solution (3.5 mg/0.5 mL) and Kryptofix 2.2.2. (15.0 mg) in acetonitrile (1.0 mL) and transferred to the reaction vessel. The aqueous [^18^F]F-solution was gradually dried at 60–120°C for 15–30 min under N_2_ flow to remove the water and acetonitrile by azeotropic drying. After complete elimination of the solvent, a solution containing the precursor (3-((2-((1E,3E)-4-(5-(methylamino)pyridin-2-yl)buta-1,3-dien-1-yl)benzo[d]thiazol-6-yl)oxy)-2-((tetrahydro-2H-pyran-2-yl)oxy)propyl-4methylbenzenesulfonate) (2.0 mg) in anhydrous DMSO (0.4 mL) was added. The mixture was heated at 120°C for 15 min. Following ^18^F-fluorination, hydrochloric acid solution (4 M, 0.5 mL) was added to the reaction vessel, and the reaction mixture was heated and maintained at 100°C for 10 min. After deprotection, sodium acetate solution (1 M, 2.0 mL) was added to the reaction vessel to neutralize the acid. After cooling to room temperature, the mixture was purified by high-performance liquid chromatography (HPLC) using a Mediterranea Sea C18 column (10 mm × 250 mm, 5 μm) as the stationary phase and acetonitrile/50-mM ammonium acetate (40/60) as the mobile phase under isocratic conditions (flow rate = 5 mL/min). The desired fraction (retention time = 25 min) was collected into a separated vessel, diluted with a sodium ascorbate solution (3% w/v, 25 mL), and reformulated using a C-18 light cartridge (Sep-Pak® Light, Waters, Milford, MA, USA). The cartridge containing florzolotau was eluted with pure ethanol (1.5 mL) into the final vial. Chemical and radiochemical purity were determined by radio-HPLC (1200 Series, Agilent, California, USA) equipped with a radio-detector (Gabi, Elysia-Raytest, Germany) and an ultraviolet (UV)-detector (Agilent, California, USA) (wavelength set at 365 nm) connected in series. An Agilent Eclipse XBD-C18 (4.6 mm × 150 mm, 5 μm) was used as the stationary phase, and methanol/5-mM ammonium acetate (70/30) as the mobile phase at a flow rate of 1 mL/min (retention time = 4.6 min). The HPLC method used was: 0 min (70/30) → 8 min (70/30) → 15 min (95/5) → 17 min (95/5) → 17.5 min (70/30) → 20 min (70/30). The identity of the desired tracer was confirmed by coelution with the reference standard. Decay-corrected radiochemical yield was 9.7% (total synthesis time: 70 min). Radiochemical purity was 96% and a molar activity of 104.4 GBq/μmol was obtained at the end of the synthesis.

### 2.9 Autoradiography

Autoradiography experiments with the radiotracer florzolotau were used to quantify the effect of rmTBI on brain neurofibrillary tangle (NFT) load. Since cellular tau accumulation in this animal model has already been shown by immunohistochemistry (McAteer et al., 2016), we chose to perform autoradiographic experiments since they allow direct quantification of NFTs. We used the florzolotau tracer, which has been already used to determine NFTs post-mortem in the brain of CTE patients (Varlow and Vasdev, 2023). At the end of the experiments, all animals were euthanized with an overdose of chloral hydrate (800 mg/kg in saline, i.p.), and then brains were taken out, placed into OCT compound (Tissue-Tek®, Sakura Finetek, Germany), and fast frozen in liquid nitrogen. Brains were sliced in coronal sections of 16-μm thickness between bregma coordinates −2 and − 4 mm (Paxinos and Watson, 2007) using a cryostate (CM3050 S, Leica Biosystems, Germany), mounted on Superfrost™ Plus adhesion microscope slides (Epredia, Microm International, Germany) and stored at −20°C until further processing. For autoradiography, brain slices of bregma −3.0 ± 0.2 mm were selected, thawed, and preincubated for 15 min in Tris–HCl buffer (50 mM, pH 7.4, supplemented with 1-mM MgCl_2_, 1 mM CaCl_2_, 2 mM KCl, and 1% of bovine serum albumin) at room temperature. Subsequently, the slices (triplicates, 3 brain slices per animal) were incubated in a 200-ml vessel containing 0.01GBq of florzolotau (0.5 nM) in buffer solution for 30 min at room temperature under gentle stirring. After incubation, the slices were removed from the bath, washed for 10 min in ice-cold buffer (50 mM Tris–HCl, pH 7.4, 4°C), and dipped once in ice-cold ultrapure water. After drying over a heating plate (40°C), the slices were exposed to a phosphor-sensitive plate for 5 min, and the plate was scanned in a phosphor imager (Amersham Typhoon 5, GE, USA) at the highest resolution (10 μm). This procedure was performed in two batches due to the number of samples, using the same radiotracer synthesis. A slide containing a control tissue (7-week-old female brain homogenate) was added for normalization on each phosphor plate. Image quantification was performed using PMOD software version 4.1 (PMOD Technologies LLC, Zürich, Switzerland). A whole brain contour was drawn on each brain slice, and the optical density (OD) was calculated. For regional analyses, contours were drawn for both the sections’ hippocampus and the parietal cortex, and OD was also calculated. For data analysis, the OD of each slice was divided by the OD from the respective control tissue, and the ratio of OD_tissue_/OD_control_ was calculated. The mean value from the triplicates was used for statistical analysis. The experimenter was blinded to the experimental groups.

### 2.10 Statistical analysis

All statistical analyses were performed with the Statistical Package for the Social Sciences (SPSS) software version 22.0 (IBM SPSS Statistics for Windows, Armonk, NY, USA), and all graphs were created in GraphPad Prism (version 8.1, GraphPad, Massachusetts, USA). As used before, the generalized estimating equation (GEE) statistical model was used to analyse multifactorial longitudinal and non-longitudinal data (Moraga-Amaro et al., 2022). This model was chosen because it can achieve higher statistical power in datasets with both complete and missing data, and also when the sample size is small, compared to statistical tests of analysis of variance (ANOVA)-type (Ma et al., 2012). For these analyses, the main effect of the factors “*injury*” (sham or rmTBI), “*sex*” (female or male), and “*time point*” were used. Two different pairwise comparison analyses were performed: (1) Injury effects (pooled/grouped data from males and females) and (2) sex-dependent effects, using the factor ‘*injury*’ and ‘*injury and sex*’, respectively for non-longitudinal data. OFT distance travelled, OFT time in centre, OFT number of entries to centre zone, Y-maze time spent in novel arms, Y-maze entries to novel arms, and autoradiography results were analysed as non-longitudinal data. For longitudinal data, the factor “time point” was added to each combination, being the injury effects and sex-dependent effects calculated by “*injury and time point*” and “*injury and sex time point*” respectively. Short-term weight gain, time-to-right, NSS, SPT, p-tau in plasma, and NSE in plasma were analysed as longitudinal data. Wald chi-square (*χ*^2^) and degrees of freedom (*df*) tests are presented for each primary effect value. In addition, correlation analyses were performed using Spearman’s test and expressed as correlation coefficient (*r*). Results from all comparisons are described as mean ± standard deviation (SD). Significance was reached when *p* < 0.05.

## 3 Results

### 3.1 RmTBI induced short-term weight gain decrease and increased time-to-right

**Figure 2:**
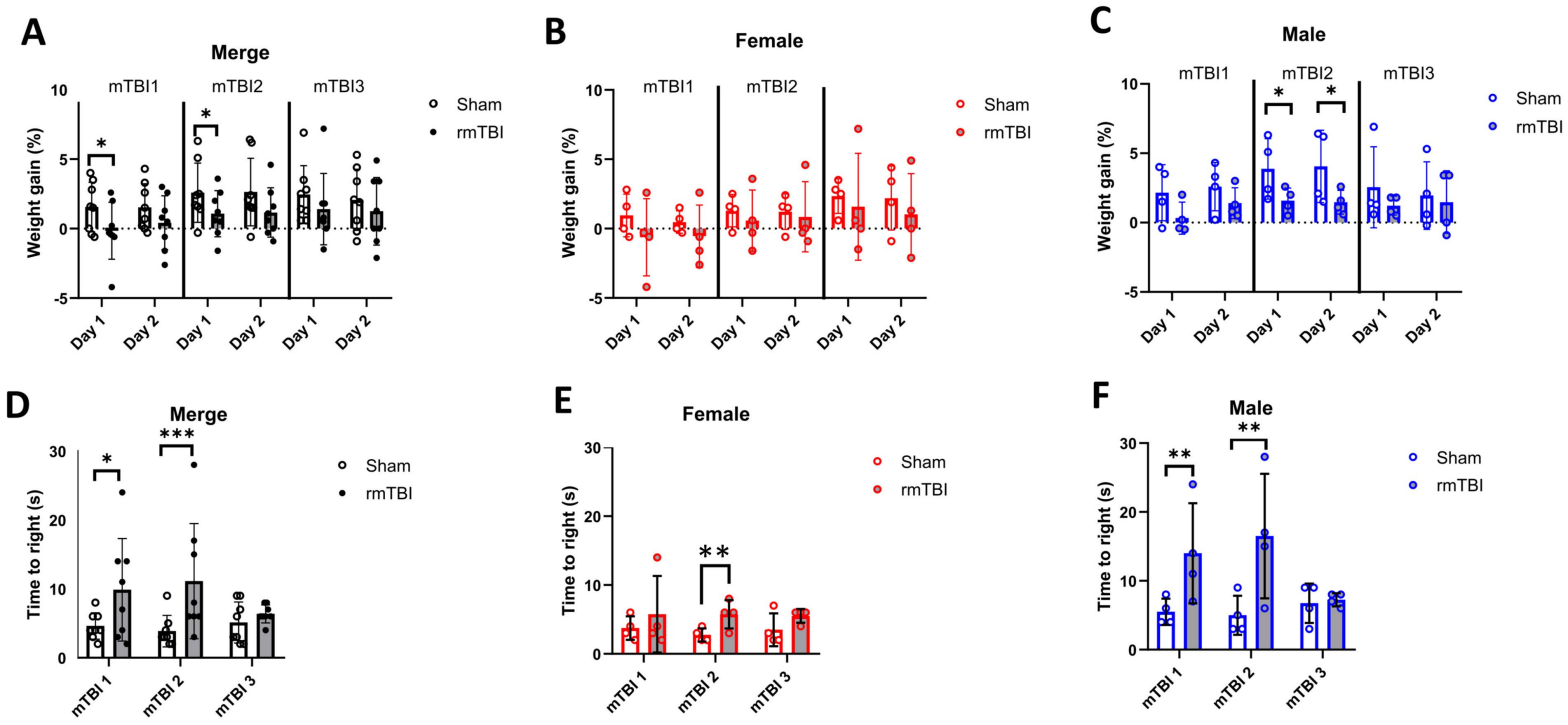
Immediate effects of mTBI surgeries. **(A–C)** Changes in weight gain on days 1 and 2 after each mTBI/Sham surgery for merged data of female and male rats, respectively. **(D–F)** Measured time-to-right when waking up from anaesthesia after each mTBI/Sham surgery for merged data, female, and male rats, respectively. Data are shown as mean ± SD. Statistically significant differences are indicated by asterisks: *p < 0.05; **p < 0.01; ***p < 0.001. Merge refers to pooled data of male and female rats.

Short-term weight changes due to rmTBI were calculated for days 1 and 2 of each of the three mTBI surgeries, using the weight before each surgery as baseline (Figures 2A–C). No significant main effects were found for *injury* (*χ*^2^ = 3; *df* = 1; *p* = 0.101) and *sex* (*χ*^2^ = 2; *df* = 1; *p* = 0.155) factors alone. The injury effects over *time* showed a significant effect (*χ*^2^ = 679; *df* = 11; *p* < 0.001). Injury induced a reduction in body weight on both day 1 after the first (Sham = 1.6 ± 1.8, rmTBI = −0.2 ± 2.0, *p* = 0.046) and second rmTBI (Sham = 2.6 ± 2.1, rmTBI = 1.1 ± 1.7, *p* = 0.045) surgery (Figure 2A). No other time points were found to be statistically different (*p* > 0.05 for all time points). Analysis of sex-dependent effects over *time* (*χ*^2^ = 3,136,095; *df* = 12; *p* < 0.001) showed that females did not show body weight gain changes (Figure 2B, *p* > 0.05), while males showed a significant decrease in body weight gain on days 1 and 2 after the second rmTBI (day 1: Male sham = 3.9 ± 2.2, Male rmTBI = 1.6 ± 0.9, *p* = 0.025; day 2: Male sham = 4.1 ± 2.6, Male rmTBI = 1.5 ± 0.9, *p* = 0.031), as shown in Figure 2C. Long-term weight changes due to rmTBI were not found (Supplementary Figure 1).

Time-to-right was measured for all animals after each rmTBI to assess acute neurological impairment. A general effect of both *sex* (*χ*^2^ = 17; *df* = 1; *p* < 0.001) and *injury* (*χ*^2^ = 17; *df* = 1; *p* < 0.001) was found. The same effect was found when comparing the effect of *injury* over *time* (*χ*^2^ = 25; *df* = 5; *p* < 0.001), where a significant increase in the time-to-right after anaesthesia was observed both after first (mTBI1: Sham = 4.6 ± 1.9, rmTBI = 9.9 ± 7.4, *p* = 0.011) and second (mTBI2: Sham = 3.8 ± 2.3, rmTBI = 11.1 ± 3.0, *p* < 0.001) mTBI (Figure 2D). When analysing the sex-dependent effect over *time* (*χ*^2^ = 226; *df* = 5; *p* < 0.001), only female rats (Figure 2E) showed an increase after mTBI2 (Sham = 2.8 ± 1.0, rmTBI = 5.8 ± 2.1, *p* = 0.002). On the contrary, male animals showed the same pattern (Figure 2F), with increased time-to-right after mTBI1 (Sham = 5.5 ± 1.9, rmTBI = 14.0 ± 7.3, *p* = 0.009) and mTBI2 (Sham = 5.0 ± 2.8, rmTBI = 16.5 ± 9.0, *p* = 0.008). No other statistical differences were found for the rest of the comparisons.

### 3.2 Neurological impairments due to rmTBI were long-lasting

**Figure 3:**
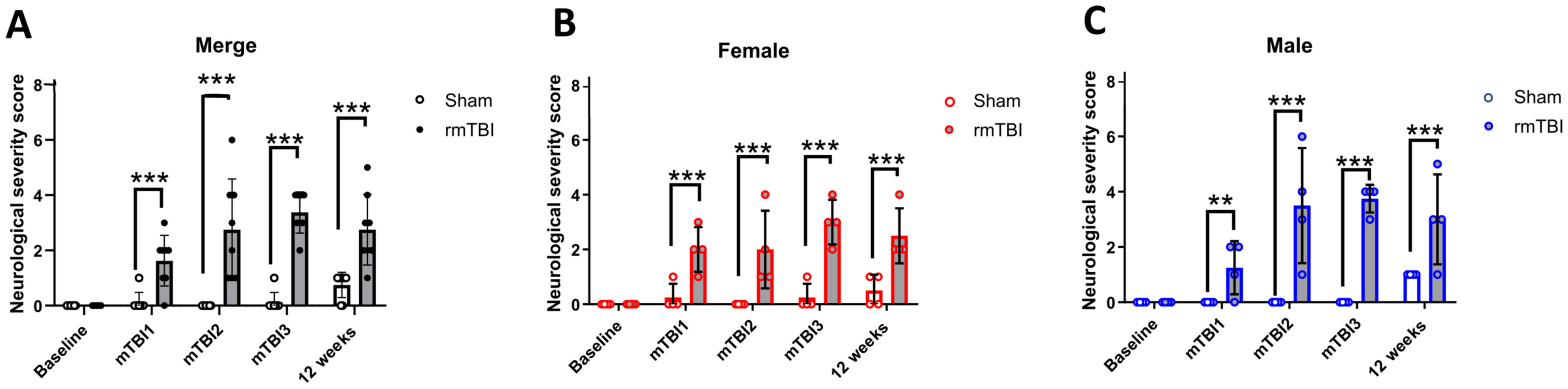
Acute and long-term neurological changes after mTBI/rmTBI. **(A–C)** Neurological severity score changes due to mTBI/Sham surgery and at 12 weeks after rmTBI of merged data of female and male rats, respectively. Data are shown as mean ± SD. **Statistically significant when *p* < 0.01; ***Statistically significant when *p* < 0.001. Merge refers to pooled data of male and female rats.

The NSS was used to assess the neurological impairments due to rmTBI over time. While *injury* showed to be influencing the NSS (*χ*^2^ = 120; *df* = 1; *p* < 0.001), this was not *sex-*dependent (*χ*^2^ = 1; *df* = 1; *p* = 0.248). When comparing the effects of injury over time (*χ*^2^ = 2,615; *df* = 6; *p* < 0.001), an increase in the NSS after each mTBI was found (mTBI1: Sham = 0.1 ± 0.4, rmTBI = 1.6 ± 0.9, *p* < 0.001; mTBI2: Sham = 0.0 ± 0.0, rmTBI = 2.8 ± 1.8, *p* < 0.001; mTBI3: Sham = 0.1 ± 0.4, rmTBI = 3.4 ± 0.7, *p* < 0.001), which lasted until 12 weeks after the last mTBI (12 weeks: Sham = 0.8 ± 0.4, rmTBI = 2.8 ± 1.3, *p* < 0.001; Figure 3A). Sex-dependent effects over time (*χ*^2^ = 4,708; *df* = 8; *p* < 0.001) showed the same effect in both females (mTBI1: Sham = 0.3 ± 0.5, rmTBI = 2.0 ± 0.8, *p* < 0.001; mTBI2: Sham = 0.0 ± 0.0, rmTBI = 2.0 ± 1.4, *p* = 0.001; mTBI3: Sham = 0.3 ± 0.5, rmTBI = 3.0 ± 0.8, *p* < 0.001; 12 weeks: Sham = 0.5 ± 0.6, rmTBI = 2.5 ± 1.0, *p* < 0.001) and males (mTBI1: Sham = 0.0 ± 0.0, rmTBI = 1.25 ± 1.0, *p* = 0.003; mTBI2: Sham = 0.0 ± 0.0, rmTBI = 3.5 ± 2.1, *p* < 0.001; mTBI3: Sham = 0.0 ± 0.0, rmTBI = 3.8 ± 0.5, *p* < 0.001; 12 weeks: Sham = 1.0 ± 0.0, rmTBI = 3.0 ± 1.6, *p* = 0.005), respectively (Figures 3B,C). No differences were found in any of the baseline comparisons.

### 3.3 Anhedonic-like behaviour due to rmTBI was found in male but not female rats

**Figure 4:**
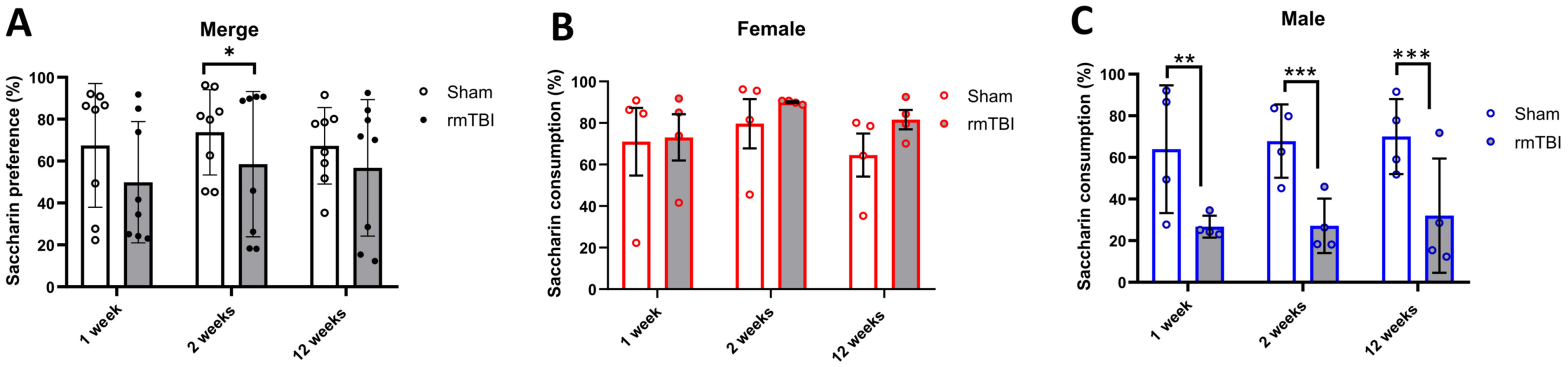
Acute and long-term changes in anhedonic-like behaviour due to rmTBI. **(A–C)** Changes in saccharin consumption at 1-, 2-, and 12 weeks after rmTBI for merged data of female and male rats, respectively. Data are shown as mean ± SD. *Statistically significant when *p* < 0.05; **Statistically significant when *p* < 0.01; ***Statistically significant when *p* < 0.001. Merge refers to pooled data of male and female rats.

To determine possible anhedonic-like behaviour, the SPT was used to measure changes in saccharin consumption at 1, 2, and 12 weeks after rmTBI. Both *injury* (*χ*^2^ = 4; *df* = 1; *p* = 0.046) and *sex* (*χ*^2^ = 16; *df* = 1; *p* < 0.001) factors showed an influence on SPT changes. At the first level, there was an effect of *injury* over *time* (*χ*^2^ = 13; *df* = 5; *p* = 0.025) and a protracted reduction in saccharin consumption due to rmTBI (Figure 4A) was found after 2 weeks (Sham = 73.8% ± 20.4, rmTBI = 58.6% ± 34.7, *p* = 0.030). This effect was not observed after 1 week or 12 weeks. When comparing the specific *sex*-dependent effect (*χ*^2^ = 33,658; *df* = 11; *p* < 0.001), a significant effect of sex was found. While female rats did not show significant differences at any time point (Figure 4B, *p* > 0.05), male rats showed a significant decrease (Figure 4C) in saccharin consumption at all time points (1 week: Sham = 64.0% ± 30.7, rmTBI = 26.7% ± 5.3, *p* = 0.006; 2 weeks: Sham = 67.8% ± 17.6, rmTBI = 27.2% ± 13.1, *p* < 0.001; 12 weeks: Sham = 70.0% ± 18.0, rmTBI = 32.0% ± 27.5, *p* = 0.007).

### 3.4 RmTBI induced protracted anxiety-like behaviour in rats without affecting locomotion

Using the OFT, locomotion and anxiety-like behaviour were evaluated, represented as distance travelled and avoidance of open spaces (time spent in the centre of the arena and number of transitions of periphery/centre).

**Figure 5:**
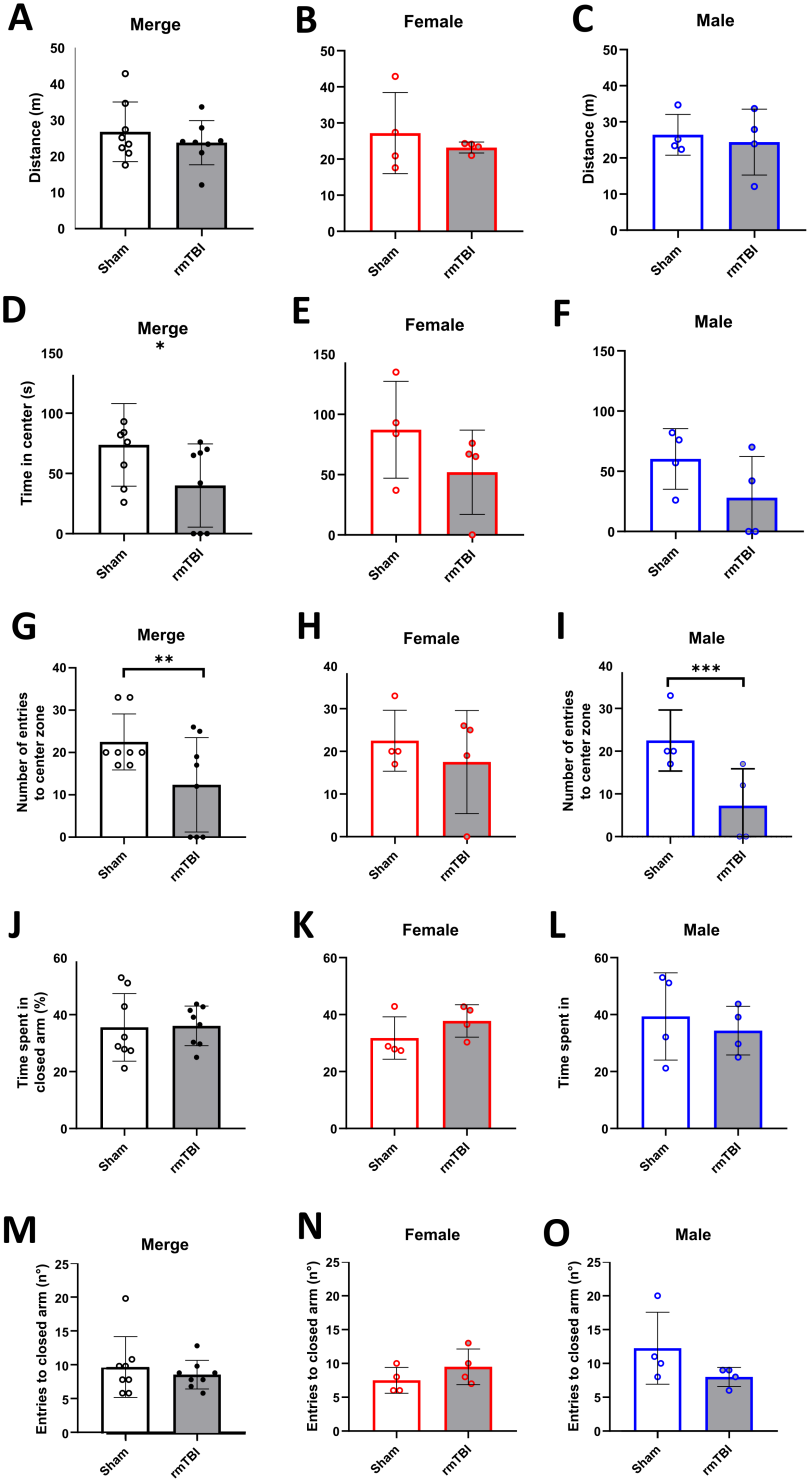
Anxiety-like behaviour and spatial memory long-term changes after rmTBI. **(A–C)** Distance travelled in the OFT of merged data of female and male rats, respectively. **(D–F)** Time spent in the centre of the OFT of merged data of female and male rats, respectively. **(G–I)** Number of entries to the centre of the OFT of merged data of female and male rats, respectively. **(J–L)** Time (in %) spent in the closed arm of the Y-Maze during the test phase of merged data of female, and male rats, respectively. **(M–O)** Number of entries to the closed arm of the Y-maze during the test phase of merged data of female and male rats, respectively. Data are shown as mean ± SD. *Statistically significant when *p* < 0.05; **Statistically significant when *p* < 0.01; ***Statistically significant when *p* < 0.001. Merge refers to pooled data of male and female rats.

When analysing changes in locomotion, no main effects of *sex* (*χ*^2^ = 0; *df* = 1; *p* = 0.950) or *injury* alone (*χ*^2^ = 1; *df* = 1; *p* = 0.373) were found. No injury effect was found (Figure 5A, *p* = 0.373). Moreover, when analysing sex-dependent effects (*χ*^2^ = 2; *df* = 3; *p* = 0.517), rmTBI affected neither female (Figure 5B, *p* = 0.415) nor male (Figure 5C, *p* = 0.663) rats’ locomotion.

In the specific case of the time in the centre, a main effect of *injury* (*χ*^2^ = 5; *df* = 1; *p* = 0.022), but not *sex* (*χ*^2^ = 3; *df* = 1; *p* = 0.084) was found. An injury effect was found when rmTBI resulted in a decrease in the total time animals spent in the centre of the arena (Figure 5D, Sham = 73.8 ± 34.3, rmTBI = 40.0 ± 34.6, *p* = 0.022). However, this effect was not seen when analysing the sex-dependent effects (*χ*^2^ = 7; *df* = 3; *p* = 0.072), meaning that a lack of effect was observed in both female (Figure 5E, *p* = 0.258) and male (Figure 5F, *p* = 0.08) rats.

The number of entries to the centre zone showed a main effect of *injury* (*χ*^2^ = 7; *df* = 1; *p* = 0.007), but not *sex* (*χ*^2^ = 2; *df* = 1; *p* = 0.156). Injury effect analysis showed a significant decrease in the entries to the centre in the rmTBI group (Figure 5G: Sham = 22.5 ± 6.6, rmTBI = 12.4 ± 11.2, *p* = 0.007). Sex-dependent analysis (*χ*^2^ = 13; *df* = 3; *p* = 0.003) revealed that this decrease was not present in female rats (Figure 5H, *p* = 0.410), but was found in males (Figure 5I: Sham = 22.5 ± 7.1, rmTBI = 7.3 ± 8.6, *p* < 0.001).

### 3.5 RmTBI did not affect spatial memory

The Y-maze test was used to determine possible impairment in spatial memory. In this test, the time animals spent in the novel arm and the number of entries to the novel arm were measured.

There was no main effect of *sex* (*χ*^2^ = 0; *df* = 1; *p* = 0.628) in the time the animals spent in the novel arm. The same was found for *injury* (*χ*^2^ = 0; *df* = 1; *p* = 0.905), where rmTBI showed no effect (Figure 5J, Sham = 35.6 ± 11.9, rmTBI = 36.1 ± 7.0, *p* = 0.905). When analysing the sex-dependent effects (*χ*^2^ = 3; *df* = 3; *p* = 0.454), both female (Figure 5K, Sham = 31.8 ± 7.4, rmTBI = 37.8 ± 5.7, *p* = 0.139) and male (Figure 5L, Sham = 39.4 ± 15.3, rmTBI = 34.4 ± 8.5, *p* = 0.513) rats lacked a significant effect of rmTBI.

There was no main effect of *sex* (*χ*^2^ = 1; *df* = 1; *p* = 0.241) in the number of entries to the novel arm. The same lack of effect was found for the *injury* factor alone (*χ*^2^ = 1; *df* = 1; *p* = 0.417), showing no effect of rmTBI injury in the total number of entries (Figure 5M, Sham = 9.9 ± 4.5, rmTBI = 8.8 ± 2.1, *p* = 0.417). Results from sex-dependent analysis were similar (*χ*^2^ = 5; *df* = 3; *p* = 0.158), showing no changes in the total number of entries of both females (Figure 5N, Sham = 7.5 ± 1.9, rmTBI = 9.5 ± 2.6, *p* = 0.157) and males (Figure 5O, Sham = 12.3 ± 5.3, rmTBI = 8.0 ± 1.4, *p* = 0.074).

### 3.6 Short-but not long-term changes in blood biomarkers due to rmTBI

**Figure 6:**
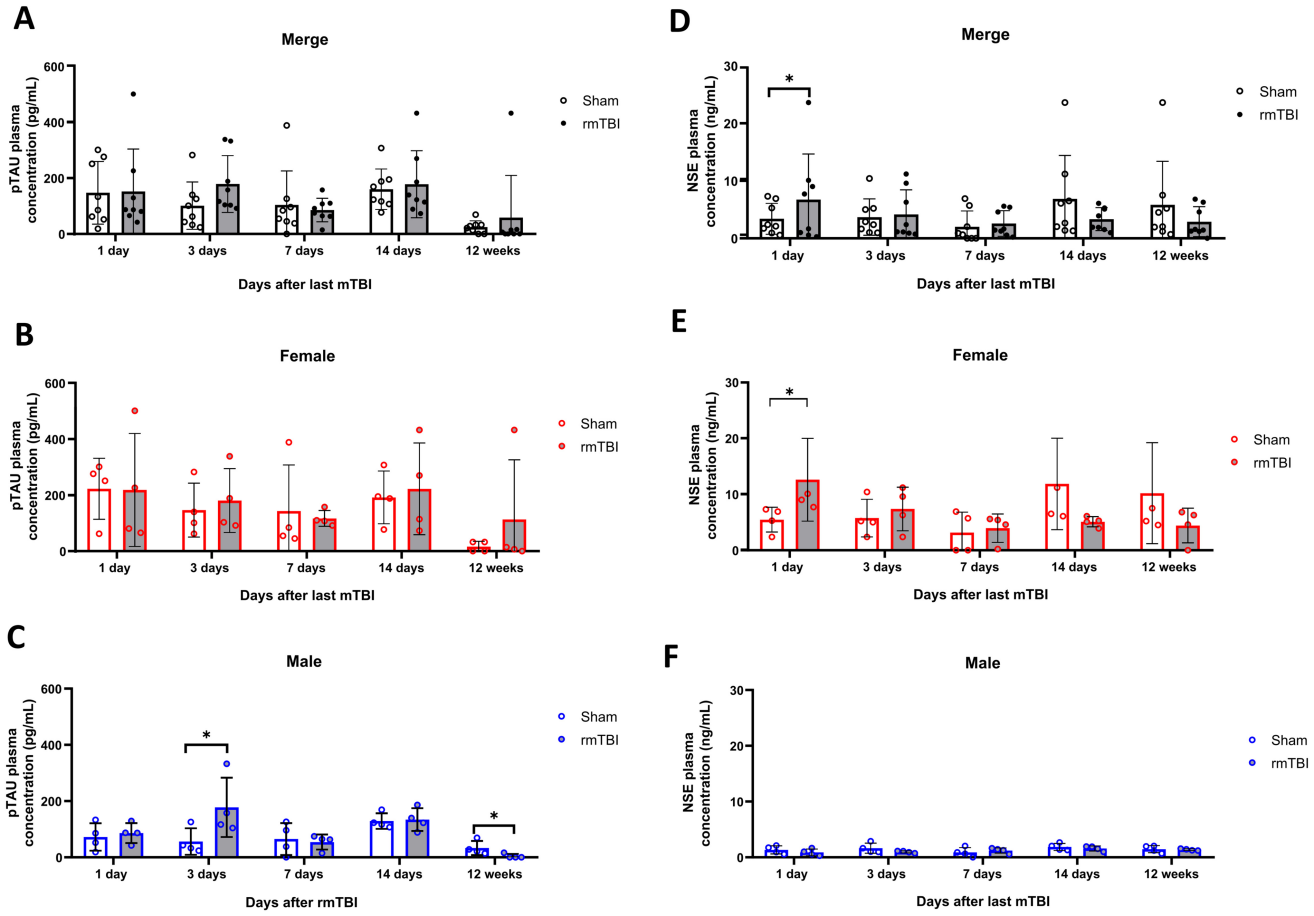
Acute and long-term changes in blood biomarkers after rmTBI. **(A–C)** Plasma p-tau measurements at days 1, 3, 7, and 14 and at 12 weeks after rmTBI/Sham of merged data of female and male rats, respectively. **(D–F)** Plasma NSE measurements at days 1, 3, 7, and 14, and at 12 weeks after rmTBI/Sham of merged data of female and male rats, respectively. Data are shown as mean ± SD. *Statistically significant when *p* < 0.05. Merge refers to pooled data of male and female rats.

Plasma changes of both p-tau and NSE were analysed at different time points as early biomarkers for rmTBI. While a main effect of *sex* (*χ*^2^ = 5; *df* = 1; *p* = 0.021) in plasma p-tau was found, no effects of *injury* (*χ*^2^ = 1; *df* = 3; *p* = 0.480) were observed. Analysis of the injury effect over time (*χ*^2^ = 108; *df* = 9; *p* < 0,001) showed no significant differences due to rmTBI injury at any time point measured (Figure 6A, *p* > 0.05 for all time points). On the contrary, when observing the sex-dependent results (*χ*^2^ = 119; *df* = 1; *p* < 0.001), although female rats showed no significant differences (Figure 6B, *p* > 0.05), male rats displayed a significant increase on day 3 after rmTBI (Figure 6C, Sham = 56.5 ± 47.0, rmTBI = 177.8 ± 105.6, *p* = 0.015). No other pairwise comparison was significant.

Similar to p-tau results, a main effect of *sex* (*χ*^2^ = 287; *df* = 12; *p* < 0.001), but not *injury* alone (*χ*^2^ = 1; *df* = 1; *p* = 0.427) was found for NSE. When observing the injury effects over time (*χ*^2^ = 3,609; *df* = 9; *p* < 0.001) an increase in blood NSE was seen at day 1 after rmTBI (Figure 6D, Sham = 3.4 ± 2.7, rmTBI = 6.7 ± 7.9, *p* = 0.048). This, however, did not persist for the rest of the measured time points (*p* > 0.05 for all time points). When analysing the sex-dependent results (χ^2^ = 5,384; *df* = 12; *p* < 0.001), a significant increase in serum NSE at day 1 was observed in female rats (Figure 6E, Sham = 5.5 ± 2.2, rmTBI = 12.6 ± 7.4, *p* = 0.033), which did not persist over the following days (*p* > 0.05). Male rats did not exhibit significant changes at any time analysed (Figure 6F, *p* > 0.05 for all time points).

### 3.7 RmTBI induced a significant increase in NFT load in the brain only in males

**Figure 7:**
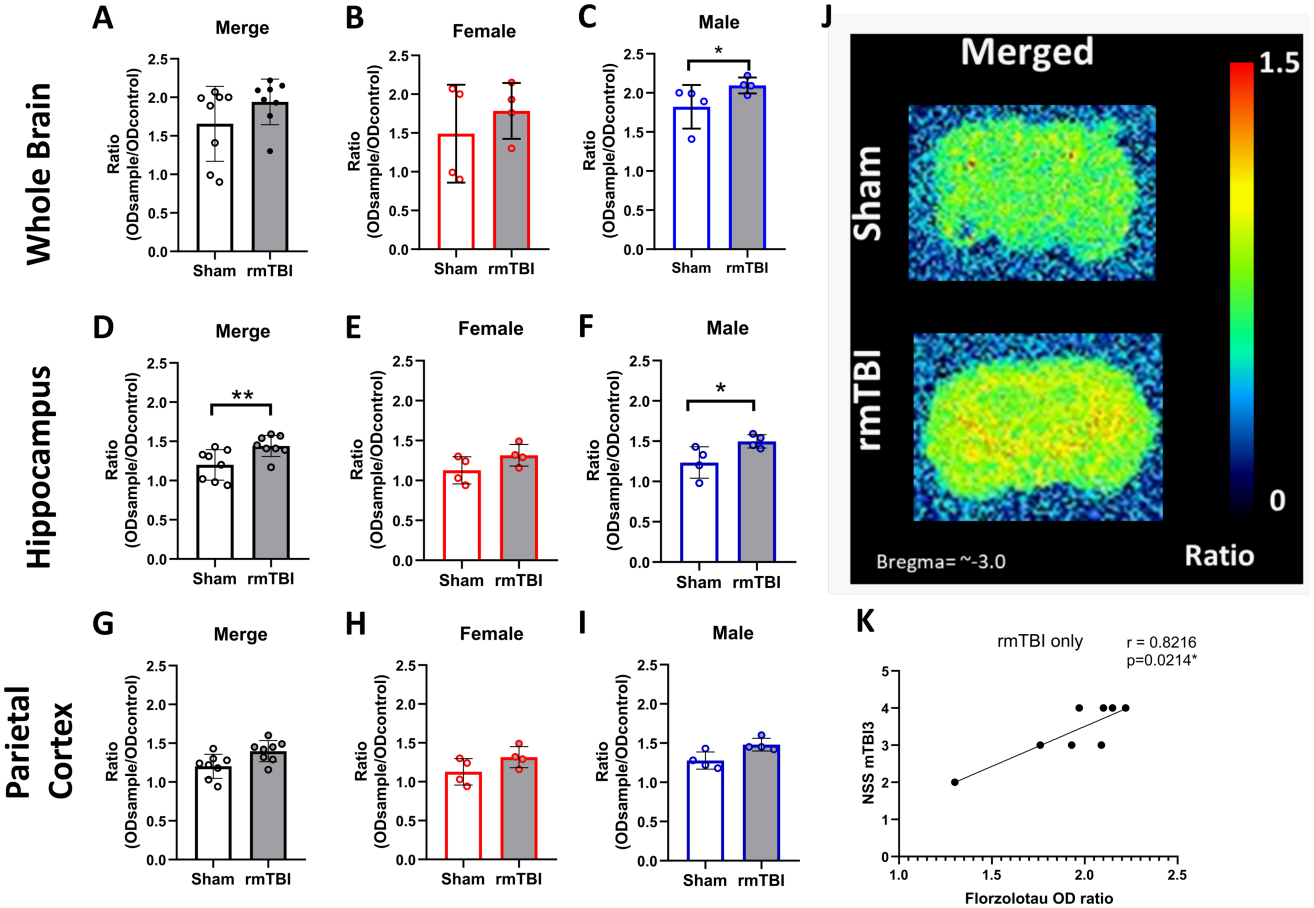
Long-term changes in brain p-tau after rmTBI. **(A–C)** Changes in florzolotau binding in rmTBI/Sham whole brain slices of merged data of female and male rats, respectively. **(D–F)** Changes in florzolotau binding in rmTBI/Sham in the hippocampus of merged data of female and male rats, respectively. **(G–I)** Changes in florzolotau binding in rmTBI/Sham parietal cortex of merged data of female and male rats, respectively. **(J)** Representative figures of florzolotau autoradiography. **(K)** Correlation analysis of florzolotau binding in the brain and NSS at day 1 after rmTBI. Data are shown as mean ± SD. *Statistically significant when *p* < 0.05. Merge refers to pooled data of male and female rats.

To determine whether the rmTBI-based CTE model influences tau accumulation in the brain (NFTs), autoradiography experiments with the radiotracer florzolotau were performed in brain slices. No main effects of *sex* (*χ*^2^ = 4; *df* = 1; *p* = 0.059) or *injury* (*χ*^2^ = 3; *df* = 1; *p* = 0.095) were found. As can be seen in Figure 7A, no injury effects on florzolotau binding were found (*p* = 0.095). However, sex-dependent analyses (*χ*^2^ = 11; *df* = 3; *p* = 0.009) further revealed that, although no significant differences are found in female rats (Figure 7B, *p* = 0.349), a significant increase in brain tau accumulation was present in males (Figure 7C, Sham = 1.8 ± 0.2, rmTBI = 2.1 ± 0.1, *p* = 0.034).

Regional analyses of florzolotau binding in the hippocampus showed a significant effect of *injury* (*χ*^2^ = 10.4; *df* = 1; *p* = 0.01) and *sex* (*χ*^2^ = 17.07; *df* = 3; *p* = 0.001). An increase in florzolotau binding due to injury was found in the hippocampus of rmTBI animals (Figure 7D, Sham = 1.2 ± 0.2, rmTBI = 1.4 ± 0.1, *p* = 0.001). When analysing the effect of sex, females did not display significant differences (Figure 7E, Sham = 1.2 ± 0.2, rmTBI = 1.4 ± 0.2, *p* = 0.065), whereas males showed a significant increase due to rmTBI (Figure 7F, Sham = 1.2 ± 0.2, rmTBI = 1.5 ± 0.1, *p* = 0.04).

On the contrary, no effects of *injury* (*χ*^2^ = 3.41; *df* = 1; *p* = 0.065) or *sex* (*χ*^2^ = 5.58; *df* = 3; *p* = 0.134) were found when analysing the florzolotau binding in the parietal cortex. Injury alone did not induce significant differences (Figure 7G, Sham = 1.2 ± 0.2, rmTBI = 1.4 ± 0.1, *p* = 0.065). The same lack of significance was also found in females (Figure 7H: Sham = 1.1 ± 0.2, rmTBI = 1.3 ± 0.1, *p* = 0.068) and males (Figure 7I, Sham = 1.3 ± 0.2, rmTBI = 1.5 ± 0.1, *p* = 0.001). Additionally, a representation of the florzolotau autoradiography outcome can be found in Figure 7J.

### 3.8 Brain NFT and peripheral p-tau correlate with early neurological severity

To determine if there is a relationship between the neurological impairment and p-tau/NFT, correlation analyses were performed between NSS and both plasma and brain NFT. When analysing the correlation between brain NFT and the neurological severity score, a strong correlation between the tau marker in animals exposed to rmTBI only and the NSS after the third mTBI (Figure 7K, *r* = 0.8216, *p* = 0.021) is found. In the same line, a medium correlation is found between NSS after the first mTBI (*r* = 0.5166, *p* = 0.042) and second mTBI (*r* = 0.5553, *p* = 0.028, not visualized) and plasma p-tau concentration at 12 weeks after rmTBI. A correlation matrix with all correlation analyses performed can be found in Supplementary Figure 2.

## 4 Discussion

The present study was designed to determine the acute effects of rmTBI and long-term CTE-like condition (at 12 weeks post-rmTBI) on neurological, behavioural, and physiological outcome parameters, analysing both injury effects and their sex-dependent effects. Our results show that the applied animal model efficiently induces both acute and long-term neurological impairments that are not sex-dependent. In addition, while rmTBI also induced an acute decrease in body weight gain and an increased time-to-right, these effects were more evident in males than in females. In the same line, rmTBI-mediated anhedonic-and anxiety-like behaviours were present only in male rats. Interestingly, changes in biological biomarkers induced by rmTBI showed to be sex-dependent, being both brain and plasma p-tau significantly increased in males. In contrast, NSE was increased only in females. Further correlation analyses revealed a strong relationship between neurological impairment early after rmTBI and brain tau at the later stage of the disease.

In the field of TBI, several animal models have been used to study the pathophysiology after both single and repetitive TBI. Given the fact that we wanted to study the pathophysiology of CTE, we used the already validated rat model by McAteer and colleagues, which is an accelerated non-penetrating model of rmTBI, mimicking head projectile impacts in contact sports (McAteer et al., 2016). In this regard, we could confirm majority of the findings made by this group. It is important to discriminate between acute and long-term effects of rmTBI, the latter reflecting the CTE-like pathology, which, in the case of our model, could simulate the long-term neurological, behavioural, and pathophysiological features of the clinical situation. To assess the severity of our model in terms of neurological impairment, we used the revised NSS (Yarnell et al., 2016), which assesses neurological and motor impairments using a scale ranging from 0 to 20, with 20 being the highest impairment score. Our results show that injury, independent of sex, induced mild neurological impairments with a maximum score of 6. Several studies have used variants of the NSS in rodents, showing mild neurological impairments in both single mTBI models (Lecques et al., 2021; Pinkowski et al., 2023) and rmTBI models (Qin et al., 2018; Cheng et al., 2019; Jacotte-Simancas et al., 2023), which is in line with our results. However, here we showed that with the rmTBI model used, the neurological impairment lasts for at least 12 weeks, which is in contrast to other studies showing no changes at 2, 4, or 6 months after rmTBI using the same NSS (Mao et al., 2018; Huynh et al., 2020). These contradicting findings highlight the specificity and limitations of each rmTBI model, as stated before (Ackermans et al., 2021).

In the last decade, CTE is associated with a higher incidence of psychiatric disorders, including depression and anxiety-related disorders (Antonius et al., 2014; Albayram et al., 2020). However, caution must be taken linking CTE with these disorders since it has been pointed out that psychiatric disorders can be case dependent, and are not necessarily a main core characteristic of CTE (Russell et al., 2020; Iverson et al., 2023). In this regard, preclinical studies can be useful in the discrimination of factors linking CTE with psychiatric disorders. Several studies conducted in different rodent models (both rats and mice) of rmTBI in both males (mostly) and females have shown that repeated events of brain trauma-induced depressive-like behaviour (Eyolfson et al., 2020; Niziolek et al., 2021; McCorkle et al., 2022; Balasubramanian et al., 2023; Wilson et al., 2023) and anxiety-like behaviour (McAteer et al., 2016; Wright et al., 2016, 2017, 2019; Bhatt et al., 2020; Huynh et al., 2020; Niziolek et al., 2021; Balasubramanian et al., 2023; Wilson et al., 2023). Still, reports where no changes in one or both parameters were detected can be found in the literature (Cheng et al., 2014; Webster et al., 2015; McCorkle et al., 2022). In this context, with the acceleration impact model used in the present study, our results support the notion that rmTBI can induce CTE-comparable depressive-and anxiety-like behaviour, but in a sex-dependent manner as male rats appear to be much more prone to develop these behavioural changes than female rats.

To avoid potential effects of short-term high calorie intake in the present study, we chose saccharin as an established non-caloric sweetener to evaluate anhedonic-like behaviour (Klein et al., 2015). There are conflicting reports regarding sex differences in the consumption of sweet solutions in healthy rats. While Tordoff et al. (2008) do not find sex differences in as many as 14 rat strains. Grimm et al. (2022) find a higher consumption of sucrose solution in females but no sex difference in saccharin consumption. Valenstein et al. (1967) report that initially, both sexes prefer saccharin over glucose which switches over time in males. Given these observations, we cannot exclude that the discriminatory power of the SCT between sham and CTE groups in the present study was influenced by different intrinsic motivations of females and males for saccharin consumption.

Sex differences in mood behaviour are a commonly discussed feature in brain diseases (Fernández-Guasti et al., 2012; Eid et al., 2019). In the clinical situation, women are more prone to develop depressive and anxiety disorders than men (Fernández-Guasti et al., 2012). A significant contribution of sex hormones to this phenomenon has been observed, with estrogen and testosterone being the main studied candidates for these effects (Fernández-Guasti et al., 2012). However, recent research suggests that there is a differential response of each biological sex to stress with regard to neural development and early life events (Fernández-Guasti et al., 2012; Altemus et al., 2014; Rubinow and Schmidt, 2019), which might also contribute to neurological disease development. In preclinical research, sex differences in behavioural test outcomes have been attributed to hormonal changes due to the oestrous cycle (Lovick and Zangrossi, 2021), while others did not find such an influence (Scholl et al., 2019). There is also evidence that the nature of the applied test influences the detection of differences between male and female rodents (Scholl et al., 2019). Since sex chromosome complement, gonadal hormones, and environment can all have a relevant influence on sex-associated differences in behavioural responses (Mir et al., 2022), the underlying mechanisms will also be multi-factorial. Nevertheless, specific changes such as sex-associated differences in stress response in the serotonergic or dopaminergic system have also been described (Dalla et al., 2008; Kamper et al., 2009), which could provide a hint for future investigations. As almost all studies on human CTE are based on male subjects, a comparison of sex-dependent effects in our study to the clinical situation has to be postponed until such information is available.

Another important feature found in patients suffering from CTE is a progressive cognitive impairment. It has already been described in several studies that there is both acute and long-term cognitive impairment in people who practice contact sports (Marklund et al., 2021; Zhuang et al., 2022; Lennon et al., 2023; Owens et al., 2023). Rodent animal models have confirmed these findings by showing impairments in many different cognitive tests which were present for days up to weeks (Nichols et al., 2016; Wright et al., 2016, 2017, 2019; McCorkle et al., 2022; Balasubramanian et al., 2023) and months (Cheng et al., 2014; Webster et al., 2015; McAteer et al., 2016; Rubenstein et al., 2019; Huynh et al., 2020; McCorkle et al., 2022; Moro et al., 2023) after rmTBI. However, also no changes have been found (Mao et al., 2018; Bhatt et al., 2020; Pham et al., 2021). In our study, we applied the Y-maze memory test, which showed no impairments in spatial memory 12 weeks after rmTBI. Nevertheless, this does not exclude the possibility that impairments might occur in another experimental setting or at other time points. In this regard, further use of a higher number of animals, in combination with additional memory tests aiming for the same memory paradigm, is needed to confirm these findings.

The fact that CTE pathophysiology and symptomatology vary between individuals and also share some commonalities with other diseases, such as Alzheimer’s disease (Rostowsky et al., 2021), makes it difficult to find effective ways to early diagnose CTE. In this regard, some effort has been made to research changes in biomarkers for CTE diagnose. Based on the molecular pathology, Halicki and colleagues divided potential biomarkers for CTE into three different groups: biomarkers for (1) neurodegeneration, (2) neuroinflammation, and (3) microRNAs (Halicki et al., 2023). Among the biomarkers of neurodegeneration which showed significant increases in plasma were p-tau, neurofilament light polypeptide (NFL), neuron-specific enolase (NSE), and Ubiquitin C-Terminal Hydrolase L1 (UCH-L1) (Agoston et al., 2022; Mavroudis et al., 2022; Halicki et al., 2023). Here, we studied the possible plasma concentration changes of plasma p-tau and NSE after rmTBI. The injury did not induce significant changes at any time point for p-tau, but further sex-dependent analysis showed a significant increase 3 days after rmTBI in male but not in female rats. When measuring NSE, a significant increase was found 1 day after rmTBI due to injury and in female rats, when analysing sex-dependent effects. We found a rather high variability of the blood biomarker values in females, with considerable overlap of blood values of sham and mTBI rats. This might limit the translational value of predictive biomarkers. One could speculate that this might be due to the oestrous cycle or a general sex-associated difference in reaction to mTBI. In this context, our results support the notion of NSE and p-tau as early biomarkers for CTE diagnosis and should therefore be part of future studies.

Furthermore, we also measured NFTs in the brain 12 weeks after rmTBI, and found higher load in the whole brain and hippocampus of male animals exposed to rmTBI. This result aligns with what was found by McAteer and colleagues in the same animal model (McAteer et al., 2016). We found that brain NFT accumulation in the applied CTE model correlated with the NSS 1 day after rmTBI. More studies are needed to determine if the severity of neurological impairments after a concussion can predict later p-tau accumulation in the brain, thus correlating with the severity of CTE in the future.

Our results are in line with studies using both sexes, showing that males tend to be more susceptible to anxiety-and depressive-related behavioural changes (Eyolfson et al., 2020; Wilson et al., 2023). We showed here that male animals exposed to rmTBI, but not females showed increased anxiety-like behaviour in the open field, as well as anhedonic-like behaviour. In addition, we demonstrate that while males after rmTBI showed an early increase in plasma p-tau and late increase in brain p-tau, female rats showed an early increase in NSE, but no changes in brain tau. These changes in biomarkers due to TBI have been already suggested in other studies (Sass et al., 2021; Papa et al., 2023). However, more studies are needed to determine how sex leads to differences in the progression of CTE among patients.

For the interpretation of our results, some limitations should be considered. The small number of animals in the sublevel analysis is a limitation; therefore, the results of this exploratory study need to be confirmed in a study using *a priori* sample-size calculation based on the present data. However, even with a small number of animals, we still found sex-dependent differences, pointing out the relevance of the factor “*sex*” in the measured parameters. In addition, as rmTBI is clinically diverse, a single animal model will not cover all aspects of the clinical situation. There will also be relevant differences between rmTBI animal models. Moreover, in this study, we quantify NFTs using autoradiography, which does not allow us (1) to observe NFTs expression in specific cell types and (2) to identify the predominant type of tauopathy generated in our model. Regardless of their limitations, for example, the lack of repeated “subconcussive events” which are common in the history of patients, the use of animal models for the study of CTE is key since it allows to overview of the progression of the disease since the beginning and to observe the long-term effects of concussion. Even if not all results can be fully generalized, they can contribute towards a multifactorial and personalized diagnosis of CTE.

## 5 Conclusion

In this study, we show that the progression of acute rmTBI effects into CTE-comparable alterations is different for males and females, in terms of symptomatology and physiological changes. The applied rat model successfully induced neurological impairment, independent of the sex, which lasted up to 12 weeks after rmTBI. In general, males were more susceptible to changes in behaviour than females. Furthermore, this study showed for the first time the sex effect translated in the differential levels of blood NSE and p-tau found in males and females, being potential sex-dependent biomarkers to early diagnose CTE. Additionally, to our knowledge, this is the first study investigating the temporal changes of different biomarkers up to 12 weeks after rmTBI. This data altogether supports other studies showing sex differences in the progression of rmTBI and adds information about how sex affects different parameters to advance a more personalized medicine and CTE diagnosis. It highlights the importance of including animals of both sexes in future experimental studies on CTE.

## Data Availability

The original contributions presented in the study are included in the article/Supplementary material, further inquiries can be directed to the corresponding author.

## Supplementary material

SUPPLEMENTARY FIGURE 1 Effect of rmTBI on weight changes. Effects of rmTBI on the percentage of weight gain after rmTBI were calculated using day 1 as baseline (i.e., day 1 after the third mTBI surgery). **(A–C)** Changes in weight gain from week 1 to week 12 after rmTBI for merged data, female and male rats respectively. No significant differences were found between Sham and rmTBI at any timepoint (*p* > 0.05). Merge refers to pooled data of male and female rats.

SUPPLEMENTARY FIGURE 2 Correlation matrix between outcome parameters. Correlation map showing positive (in blue) and negative (in red) correlations between different parameters. SPT-2w refers to saccharin preference test 2-weeks after rmTBI. NSS-1d refers to neurological severity score at 1-day after rmTBI. NSS-12w refers to neurological severity score at 12 weeks after rmTBI. OFTloc refers to the locomotion in the open field test. OFTtic refers to the time spent in the center in the open field test. OFTent refers to the number of entries to the center of the open field test. Y-M% refers to the percentage of time the animals spent in the previously closed arm of the Y-maze. Y-Ment refers to the number of entries of the animals to the previously closed arm of the Y-maze. pTAU-3d refers to the plasma p-tau measured 3 days after rmTBI. pTAU-12w refers to the plasma p-tau measured 12-weeks after rmTBI. NSE-1d refers to the plasma NSE measured 1 day after rmTBI. NSE-12w refers to the plasma NSE measured 12 weeks after rmTBI. Color scale indicates correlation coefiicient (r) between two variables; positive correlations tend to blue while negative correlations tend to red.
