# Supplementary figures and images for: Short- and long-term changes in neurological, behavioural, and blood biomarkers following repeated mild traumatic brain injury in rats—potential biological sex-dependent effects
Source: Front Mol Neurosci. 2025 Jan 29;18:1488261. doi: 10.3389/fnmol.2025.1488261 (PMC11814444; doi:10.3389/fnmol.2025.1488261)

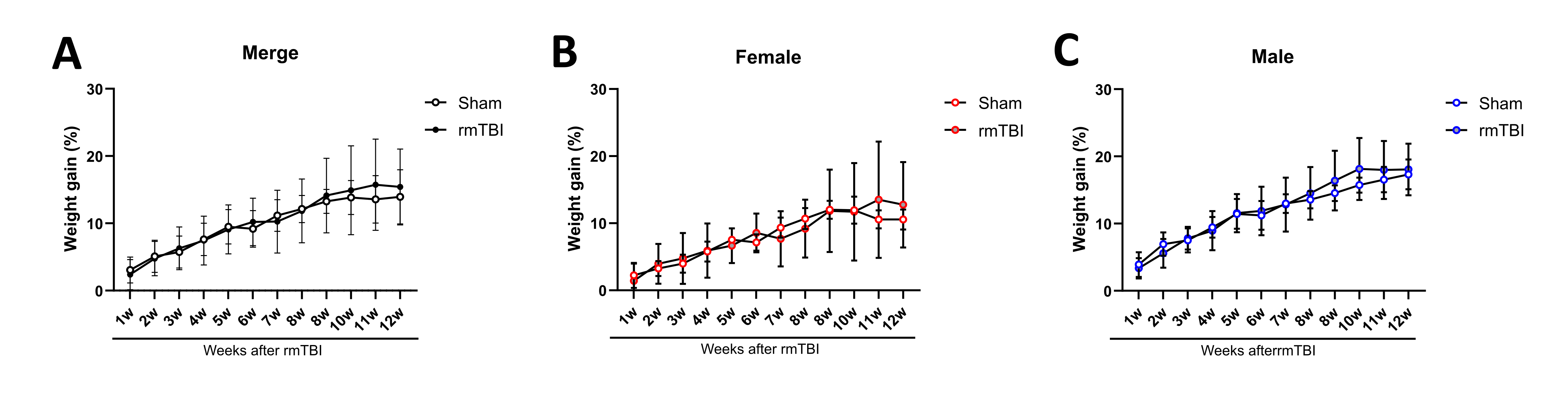

Supplement: SUPPLEMENTARY FIGURE 1 — Effect of rmTBI on weight changes. Effects of rmTBI on the percentage of weight gain after rmTBI were calculated using day 1 as baseline (i.e., day 1 after the third mTBI surgery). (A–C) Changes in weight gain from week 1 to week 12 after rmTBI for merged data, female and male rats respectively. No significant differences were found between Sham and rmTBI at any timepoint (p > 0.05). Merge refers to pooled data of male and female rats. [file Image_1.JPEG]

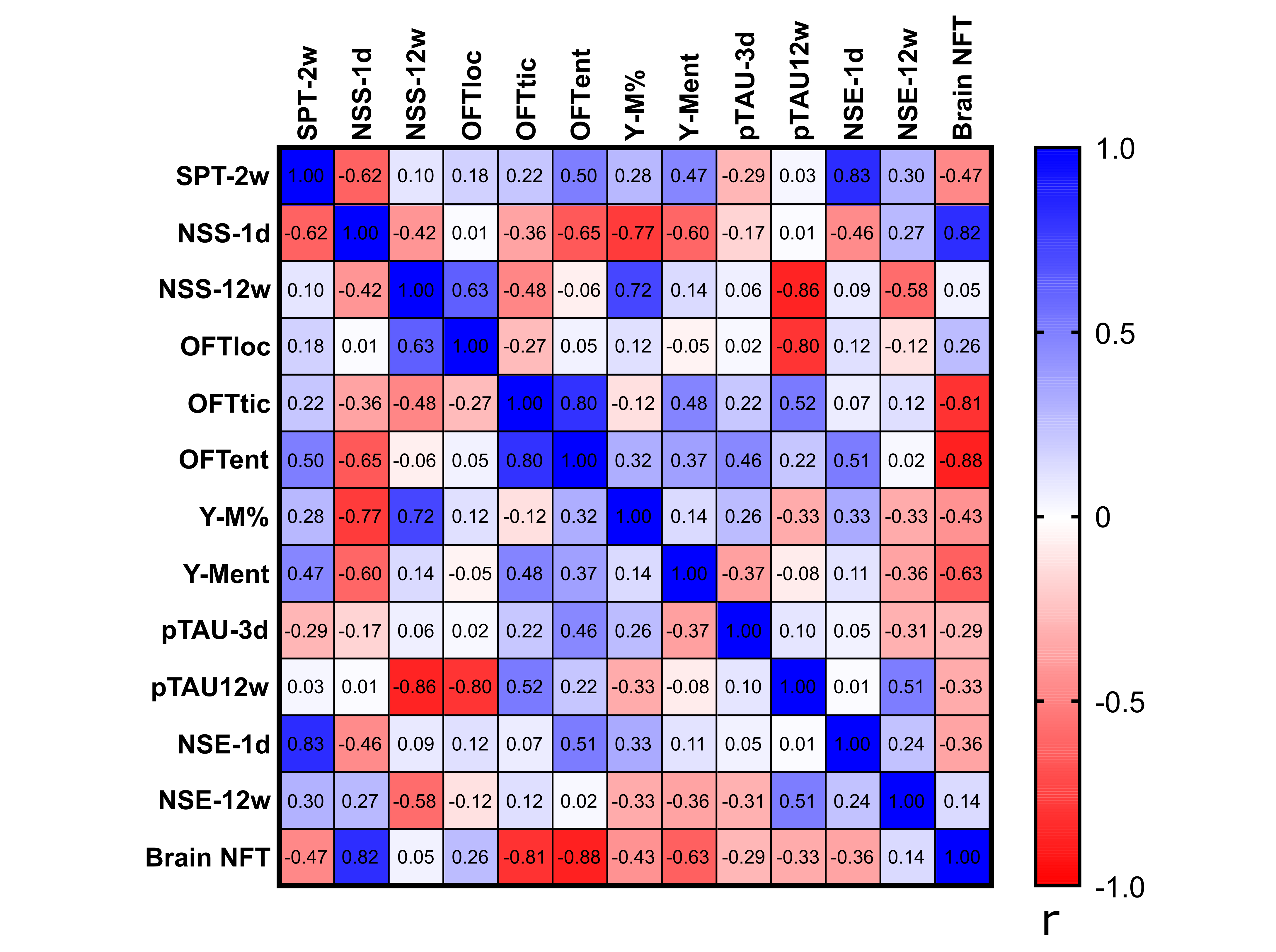

Supplement: SUPPLEMENTARY FIGURE 2 — Correlation matrix between outcome parameters. Correlation map showing positive (in blue) and negative (in red) correlations between different parameters. SPT-2w refers to saccharin preference test 2-weeks after rmTBI. NSS-1d refers to neurological severity score at 1-day after rmTBI. NSS-12w refers to neurological severity score at 12 weeks after rmTBI. OFTloc refers to the locomotion in the open field test. OFTtic refers to the time spent in the center in the open field test. OFTent refers to the number of entries to the center of the open field test. Y-M% refers to the percentage of time the animals spent in the previously closed arm of the Y-maze. Y-Ment refers to the number of entries of the animals to the previously closed arm of the Y-maze. pTAU-3d refers to the plasma p-tau measured 3 days after rmTBI. pTAU-12w refers to the plasma p-tau measured 12-weeks after rmTBI. NSE-1d refers to the plasma NSE measured 1 day after rmTBI. NSE-12w refers to the plasma NSE measured 12 weeks after rmTBI. Color scale indicates correlation coefiicient (r) between two variables; positive correlations tend to blue while negative correlations tend to red. [file Image_2.JPEG]
